# Diagnosis-specific Cumulative Incidence of Return-to-work, Resignation, and Death Among Long-term Sick-listed Employees: Findings From the Japan Epidemiology Collaboration on Occupational Health Study

**DOI:** 10.2188/jea.JE20200541

**Published:** 2022-09-05

**Authors:** Chihiro Nishiura, Yosuke Inoue, Ikuko Kashino, Akiko Nanri, Motoki Endo, Masafumi Eguchi, Takeshi Kochi, Noritada Kato, Makiko Shimizu, Teppei Imai, Akiko Nishihara, Makoto Yamamoto, Hiroko Okazaki, Kentaro Tomita, Toshiaki Miyamoto, Shuichiro Yamamoto, Tohru Nakagawa, Toru Honda, Takayuki Ogasawara, Naoko Sasaki, Ai Hori, Isamu Kabe, Tetsuya Mizoue, Seitaro Dohi

**Affiliations:** 1 Department of Safety and Health, Tokyo Gas Co., Ltd., Tokyo, Japan; 2 Department of Epidemiology and Prevention, Center for Clinical Sciences, National Center for Global Health and Medicine, Tokyo, Japan; 3 Department of Food and Health Sciences, International College of Arts and Sciences, Fukuoka Women’s University, Fukuoka, Japan; 4 Department of Public Health, Juntendo University Graduate School of Medicine, Tokyo, Japan; 5 Furukawa Electric Co., Ltd., Tokyo, Japan; 6 Fuji Electric Co., Ltd., Tokyo, Japan; 7 East Japan Works (Keihin), JFE Steel Corporation, Kanagawa, Japan; 8 OH Support, Kanagawa, Japan; 9 Azbil Corporation, Tokyo, Japan; 10 Yamaha Corporation, Shizuoka, Japan; 11 Mitsui Chemicals, Inc., Tokyo, Japan; 12 Healthplant Co., Ltd., Tokyo, Japan; 13 East Nippon Works Kimitsu Area, Nippon Steel Corporation, Chiba, Japan; 14 Hitachi, Ltd., Ibaraki, Japan; 15 Mitsubishi Fuso Truck and Bus Corporation, Kanagawa, Japan; 16 Department of Global Public Health, Faculty of Medicine, University of Tsukuba, Ibaraki, Japan; 17 Kubota Corporation, Tokyo, Japan

**Keywords:** mental disorders, neoplasms, retirement, return to work, sickness absence

## Abstract

**Background:**

While it is essential to understand how long is sufficient for return-to-work when designing paid sick-leave systems, little attempt has been done to collect cause-specific information on when and how many of sickness absentees returned to work, became unemployed, or passed away.

**Methods:**

We studied the first sick-leave episode of ≥30 consecutive days in those ≤55 years of age during 2012–2013 among employees of 11 Japanese private companies (*n* = 1,209), which were followed until 2017. Overall and disease-specific cumulative incidences of return-to-work, resignations, and deaths were estimated using competing risk analysis.

**Results:**

During the 3.5-year period (follow-up rate: 99.9%), 1,014 returned to work, 167 became unemployed, and 27 died. Overall, return-to-work occurred within 1 year in 74.9% of all absentees and in 89.3% of those who successfully returned to work. Resignation occurred within 1 year in 8.7% of all absentees and in 62.9% of all subjects who resigned. According to ICD-10 chapters, the cumulative incidence of return-to-work ranged from 82.1% for mental disorders (F00–F99) to 95.3% for circulatory diseases (I00–I99). The cumulative incidence of return-to-work due to mental disorders ranged from 66.7% in schizophrenia (F20) to 95.8% in bipolar affective disorders (F31). Death was rarely observed except for cases of neoplasms (C00–D48), of which the cumulative incidence of death reached 14.2% by 1.5 years.

**Conclusion:**

Return-to-work and resignations occurred commonly within 1 year of sick leave among long-term sickness absentees in the Japanese private companies. Our findings may assist occupational physicians and employers in developing effective social protection schemes.

## INTRODUCTION

Social protection plays a key role in ensuring income security for people of working age, which is an essential component of the well-being of employees and their families, and in achieving sustainable development goals.^1^ Income replacement during sick leave has been regarded as an essential social protection scheme; for example, the International Labour Organization recommends that their member countries implement paid sick leave as a national social protection floor.^2^

When policymakers or employers introduce or reform their sick-leave system in their countries or companies, it is essential to understand how long is sufficient for a paid sick leave that enables employees to return to work (RTW). Until now, several studies have described the outcomes experienced by those who took sick leave (ie, when and how many of them returned to work, became unemployed, or passed away).^3^^–^^5^ For example, previous studies have reported RTW experienced by those who took sick leave due to mental disorders,^6^^–^^8^ cancer,^9^^,^^10^ and musculoskeletal diseases.^7^

Despite this progress, these previous studies have some limitations. First, previous studies have focused on specific causes of sick leave, like mental disorders, instead of considering several major causes within the same study populations.^6^^–^^12^ Second, most previous studies did not consider competing risks,^6^^–^^8^^,^^10^^,^^11^ which cannot be estimated by the Kaplan-Meier estimate. There is a need to use competing risk models accounting for possible consequences of sickness absence, like RTW, resignation, and death. Third, some previous studies followed study participants for 1 year or less,^8^^,^^9^^,^^11^ which may be too short to understand the consequences experienced by most absentees. Finally, previous findings mostly came from specifically European cohorts, and research from other populations is needed to confirm the generalizability of previous findings.^3^^,^^4^

In this study, we addressed these issues by studying companies where employees can receive sick-leave benefits for 2.5 years or more and analyzing in-company administrative records of long-term sick-leave episodes with a competing risk model to describe when and how many sick-listed employees returned to work, became unemployed, or passed away among large private companies in Japan.

## METHODS

### Study population

Data were obtained from the Japan Epidemiology Collaboration on Occupational Health (J-ECOH) study cohort, which is an ongoing multicenter occupational health registry of annual health check-ups and sick leave in Japan based on administrative individual data provided by 12 companies.^13^ Of them, we studied 11 large-scale private companies (including electric machinery and apparatus manufacturing, steel, chemical, gas, nonferrous metal manufacturing, automobile and instrument manufacturing, plastic product manufacturing). Source population included approximately 80,000 employees aged 19–55 years.

The J-ECOH cohort is composed mainly of large-scale private companies; the participating companies employ occupational physicians, and their employees can receive occupational health services. This situation is not applicable to approximately 60% of Japan’s workforce who is employed in small-scale establishments with less than 50 workers that are not obligated to contract with occupational physicians.^14^ The J-ECOH cohort is also unique in that the majority of workers are male (83% of this study source population).

### Sick-leave system

In Japan, there is a lack of legislated statutory entitlements for paid sick leave, and so paid sick-leave schemes vary among companies. In the J-ECOH study, all participating companies provided paid sick-leave entitlement to their employees. The percentage of replacement for past earnings by sick pay was 100% until 18 months after sick leave, after which it ranged from two-thirds to full payment of past earnings according to companies. The maximum length of sick-leave benefits (the sum of paid annual leaves and paid sick leave) varied between 2.5 and 3.9 years (median 3 years), depending on the company. Then, the impact of the expiration of sick-leave benefits on the course of sick leaves would appear around 2.5 years or later, depending on the company. The duration of sick leaves was calculated from the initial day of work absence and presented as calendar days instead of workdays.

When using paid sick leave, the employees must apply to the company for paid sick leave with a medical certificate from the attending physician, namely, general practitioners or specialists but not occupational physicians. In the case of RTW, occupational health physicians employed by the companies assessed absentees’ work readiness and arranged planned RTW to enhance work continuity.

According to a Japanese survey on sick-leave systems conducted in 2012 (5,904 responding companies), while 91.9% of companies have introduced sick-leave systems, the distribution of available sick-leave periods in the system is reported to vary by company size.^15^ For example, 38.7% of companies with 49 or fewer full-time employees and 85.2% of companies with 1,000 or more full-time employees have a sick-leave period of one year or more. Similar to this cohort, the percentage of companies with an available sick-leave period of 2.5 years or more is 28.2% in companies with 1,000 or more full-time employees, compared to 14.5% in companies with 49 or fewer full-time employees. Therefore, it is assumed that companies participating in this cohort fall into the category of Japanese companies that have generous sick-leave systems.

### Sick-leave data

In the J-ECOH study, the administrative sick-leave records of the participating companies as a whole were collected biannually. Sick leave data obtained included the following: date of birth, sex, start and end dates of sick leave, outcome (RTW, resignation, or death), and the medically certified diagnosis for sick leave (in text style). The text of medical certificates were coded according to the International Classification of Diseases 10th revision (ICD-10) with reference to the Japanese standard disease-code master as follows.^16^ Certificates were automatically coded by text matching. Unmatched cases were independently coded by two occupational physicians (CN and CK). If they agreed, that code was adopted. If they did not agree, another occupational physician (AH) coded to break the tie. The date of death was based on death certificates in most cases, while some cases were based on information from family members. All outcomes were classified according to the diagnosis by the medical certificate at the start of sick leave.

### Inclusion criteria

Inclusion criteria for the present cohort of employees on sick leave are as follows: 1) those who took sick leaves that started during the 2 years between April 1, 2012 and March 31, 2014 and lasted 30 days or more (referred to as “long-term sick leave” according to Japanese customary definition); and 2) those who were aged 55 years or less at the beginning of the sick leave (to eliminate mandatory retirement during sick leave). If two or more sick leaves were identified for a given worker, we chose the first one to include them in the cohort. Consequently, a total of 1,209 sick leaves were included in the analysis and were followed until RTW, resignation, or death (end of follow-up: March 31, 2017).

### Exclusion criteria

We excluded those who took a long sick leave due to pregnancy-related diseases (O00–O99) for the following reasons: in the case of childbirth, absentees due to pregnancy-related diseases were removed from the framework of a sick-leave system due to successive transition to the maternal-leave system, and we had no available data for maternal leave.

### Outcome

The study outcomes were: (1) RTW, which was defined as starting to work again after taking a long-term sick-leave episode; (2) resignation; or (3) deaths of all causes, including death by suicide. There are three types of resignations among sickness absentees: voluntary turnover, turnover due to expiration of sick leave benefits, and mandatory retirement. Since the inclusion criteria used in the study had age restrictions with the intention to exclude possible mandatory retirement, we assumed that the types of resignation included in the analysis were limited to voluntary turnover and turnover due to expiration of sick leave benefits.

### Statistical analysis

Subjects were followed from 30 days after sick leave to the end of their sick-leave benefits, which varied between 2.5 and 3.9 years (median 3 years) depending on the company. While all subjects in this study took at least 30 days of sick leave, we presented the follow-up time as duration of sick leaves, which was the time from the first day of sick leave to the end of sick leave or sick-leave benefits so that the results would be easier to understand. The cumulative incidence of RTW, resignation, and death on each day of sick leave was calculated using survival analysis. Since the Kaplan-Meier estimate is not an appropriate summary of survival in the presence of competing risks,^17^ we treated RTW, resignation, and death as competing risks to calculate the cumulative incidence for each outcome using competing risk analysis with the cumulative incidence function.

We repeated the analysis for major causes of long-term sick leave (ICD-10): mental disorders (F00–F99), neoplasms (C00–D48), circulatory diseases (I00–I99), musculoskeletal diseases (M00–M99), and injuries (S00–T98).^13^ We additionally presented subgroup results of mental disorders: schizophrenia (F20), bipolar affective disorders (F31), depressive episodes (F32), other anxiety disorders (F41), and reactions to severe stress and adjustment disorders (F43). Since there was one missing case due to childbirth during sick leave for mental disorders, we treated this case as a censored case in the analysis.

All statistical analyses were performed using Stata 14 (StataCorp, College Station, TX, USA) with the user-written command ‘stcompet’.^18^

### Ethical approval

This study was approved by the Institutional Review Board of the National Center for Global Health and Medicine (NCGM-G-001140-15). This observational study was conducted with opt-out consent. Information on the implementation of the research was communicated on the internal bulletin boards of each company. While the participants did not provide written informed consent to join the study, they were allowed to refuse to participate in the study at any time. This procedure complies with the Japanese Ethical Guidelines for Epidemiological Research, which facilitates the procedure for obtaining consent in observational studies using existing data. All data were analyzed anonymously.

## RESULTS

Table 1 presents the baseline characteristics of long-term absentees. The mean age was 40.5 years and 82.6% of absentees were men, reflecting the distribution of the J-ECOH study cohort.^13^ Of 1,209 workers who took long-term sick leave, 1,014 (83.9%) returned to work, 167 (13.8%) resigned, and 27 (2.2%) died, with 1 missing case (median duration of sick leave, 98 days; range, 30–1,306 days; follow-up rate: 99.9%). No absentee remained on sick leave at the end of the observation period. Overall, RTW occurred within 1 year in 74.9% of all absentees (median time to RTW: 90 days), and 89.3% of all subjects who successfully returned to work did so within 1 year, as shown in Table 2 and Table 3. Resignation occurred within 1 year in 8.7% of all absentees (median time to resignation: 249 days), and 62.9% of all subjects who ended up resigning did so within 1 year.

**Table 1.** Baseline characteristics of 30 days or more of sick-listed employees in the J-ECOH cohort, 2012–2013

Causes of sick leave (ICD-10 codes)	*N* (% of overall)	Mean onset age, years (SD)	Men, %
Overall (A00–U99)	1,209 (100)	40.5 (9.6)	82.6
Mental disorders (F00–F99)	695 (57.5)	38.8 (9.5)	85.9
Schizophrenia (F20)	21 (1.7)	42.0 (8.0)	71.4
Bipolar affective disorders (F31)	24 (2.0)	41.9 (8.6)	91.7
Depressive episodes (F32)	418 (34.6)	39.5 (9.3)	88.8
Other anxiety disorders (F41)	40 (3.3)	37.2 (9.7)	77.5
Reaction to severe stress, ​ and adjustment disorders (F43)	108 (8.9)	35.6 (9.7)	79.6
Neoplasms (C00–D48)	113 (9.3)	45.3 (7.6)	64.6
Circulatory diseases (I00–I99)	64 (5.3)	47.8 (6.2)	89.1
Musculoskeletal diseases (M00–M99)	83 (6.9)	42.5 (8.9)	78.3
Injuries (S00–T98)	103 (8.5)	38.7 (10.2)	78.6

ICD-10, International Classification of Disease, 10^th^ revision; SD, standard deviation.

**Table 2.** Diagnosis-specific cumulative incidence and median time of return-to-work and resignation in 30 days or more of sick leave

Causes of sick leave (ICD-10 codes)	*N*	Return-to-work							Resignation						
	
		*N*	Cumulative incidence at time from sick leave, %					Median time, days	*N*	Cumulative incidence at time from sick leave, %					Median time, days
	
			0.5 yr.	1 yr.	1.5 yr.	2 yr.	3.5 yr.			0.5 yr.	1 yr.	1.5 yr.	2 yr.	3.5 yr.	
Overall (A00–U99)	1,209	1,014	63.6	74.9	78.1	80.0	83.9	90	167	5.5	8.7	9.5	10.6	13.8	249
Mental disorders (F00–F99)	695	570	56.6	70.3	73.8	76.4	82.1	118	121	6.3	10.7	11.8	13.3	17.4	259
Schizophrenia (F20)	21	14	19.1	47.6	52.4	61.9	66.7	251	6	9.5	9.5	9.5	14.3	28.6	785
Bipolar affective disorders (F31)	24	23	45.8	62.5	66.7	70.8	95.8	249	1	0.0	0.0	0.0	4.2	4.2	575
Depressive episodes (F32)	418	352	58.1	72.0	75.8	78.5	84.2	118.5	66	6.7	9.6	10.3	11.5	15.7	230
Other anxiety disorders (F41)	40	30	52.5	65.0	67.5	67.5	75.0	114.5	9	7.5	17.5	20.0	20.0	22.5	247
Reaction to severe stress, ​ and adjustment disorders (F43)	108	84	62.5	72.8	74.7	75.6	78.4	91.5	23	5.6	14.1	15.9	17.8	21.6	274
Neoplasms (C00–D48)	113	91	63.7	75.2	79.7	80.5	80.5	68	3	1.8	1.8	1.8	1.8	2.7	88
Circulatory diseases (I00–99)	64	61	79.7	85.9	90.6	90.6	95.3	59	2	0.0	0.0	0.0	1.6	3.1	865
Musculoskeletal diseases (M00–M99)	83	71	67.5	80.7	81.9	84.3	85.5	75	11	6.0	8.4	8.4	10.8	13.3	241
Injuries (S00–T98)	103	95	86.4	89.3	92.2	92.2	92.2	60	8	3.9	7.8	7.8	7.8	7.8	180

ICD-10, International Classification of Disease, 10^th^ revision.

**Table 3.** Changes in percentage of total return-to-work and resignation during sick leave

Causes of sick leave (ICD-10 codes)	Return-to-work						Resignation					
	
	*N*	Percentage of total return-to-work at time from sick leave, %					*N*	Percentage of total resignation at time from sick leave, %				
	
		0.5 yr.	1 yr.	1.5 yr.	2 yr.	3.5 yr.		0.5 yr.	1 yr.	1.5 yr.	2 yr.	3.5 yr.
Overall (A00–U99)	1,014	75.8	89.3	93.1	95.4	100.0	167	39.5	62.9	68.9	76.6	100.0
Mental disorders (F00–F99)	570	68.9	85.8	90.0	93.2	100.0	121	36.4	61.2	67.8	76.0	100.0
Schizophrenia (F20)	14	28.6	71.4	78.6	92.9	100.0	6	33.3	33.3	33.3	50.0	100.0
Bipolar affective disorders (F31)	23	47.8	65.2	69.6	73.9	100.0	1	0.0	0.0	0.0	100.0	100.0
Depressive episodes (F32)	352	69.0	85.5	90.1	93.2	100.0	66	42.4	60.6	65.2	72.7	100.0
Other anxiety disorders (F41)	30	70.0	86.7	90.0	90.0	100.0	9	33.3	77.8	88.9	88.9	100.0
Reaction to severe stress, ​ and adjustment disorders (F43)	84	81.0	94.0	96.4	97.6	100.0	23	26.1	65.2	73.9	82.6	100.0
Neoplasms (C00–D48)	91	79.1	93.4	98.9	100.0	100.0	3	66.7	66.7	66.7	66.7	100.0
Circulatory diseases (I00–I99)	61	83.6	90.2	95.1	95.1	100.0	2	0.0	0.0	0.0	50.0	100.0
Musculoskeletal diseases (M00–M99)	71	78.9	94.4	95.8	98.6	100.0	11	45.5	63.6	63.6	81.8	100.0
Injuries (S00–T98)	95	93.7	96.8	100.0	100.0	100.0	8	50.0	100.0	100.0	100.0	100.0

ICD-10, International Classification of Disease, 10^th^ revision.

**Figure 1.**
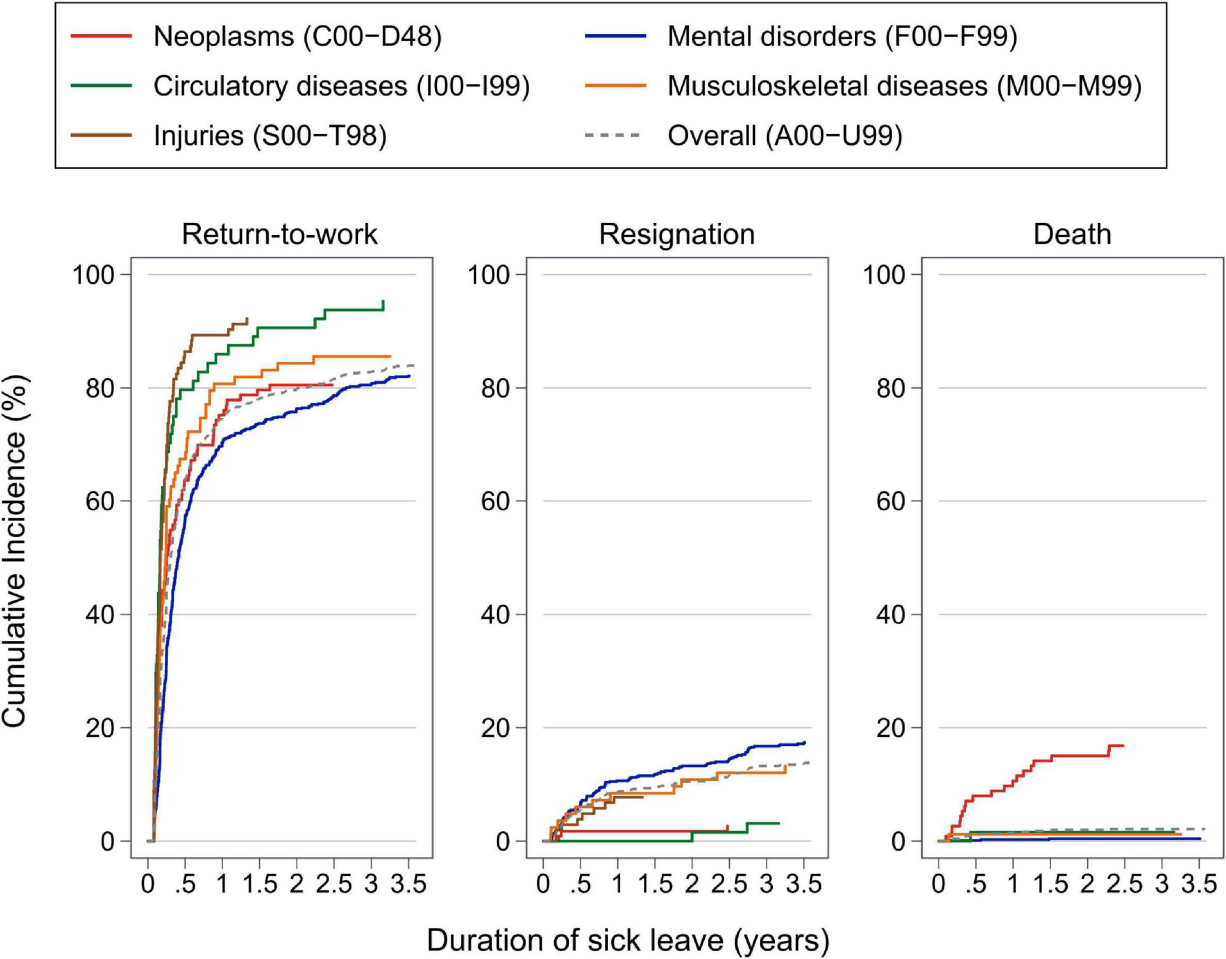
Cumulative incidence of return-to-work, resignation, and death after 30 days or more of sick leave, by major ICD-10 chapters

Figure 1 shows survival curves for the cumulative incidences of RTW, resignation, and death by ICD-10 chapters. Generally, the RTW rate increased sharply until 1 year (1-year RTW rate: neoplasms, 75.2%; mental disorders, 70.3%; circulatory diseases, 85.9%; musculoskeletal diseases, 80.7%; injuries, 89.3%) and flattened after approximately 1.5 years. According to ICD-10 chapters, the cumulative incidence of RTW ranged from 82.1% for mental disorders to 95.3% for circulatory diseases. Of the subjects who successfully returned to work by 3.5 years of sick leave, the proportion of subjects who returned to work by 1 year ranged from 85.8% in mental disorders to 96.8% in injuries. Resignation rarely occurred among absentees with neoplasms and circulatory diseases. In contrast, 17.4%, 13.3%, and 7.8% of absentees with mental disorders, musculoskeletal diseases, and injuries resigned from work before the end of sick leave, respectively. Of the subjects who resigned, 61.2% with mental disorders, 63.6% with musculoskeletal diseases, and 100% with injuries retired within 1 year. The death rate due to neoplasms reached 10.6%, 14.2%, and 16.8% by 1, 1.5, and 3.5 years of sick leave, respectively.

**Figure 2.**
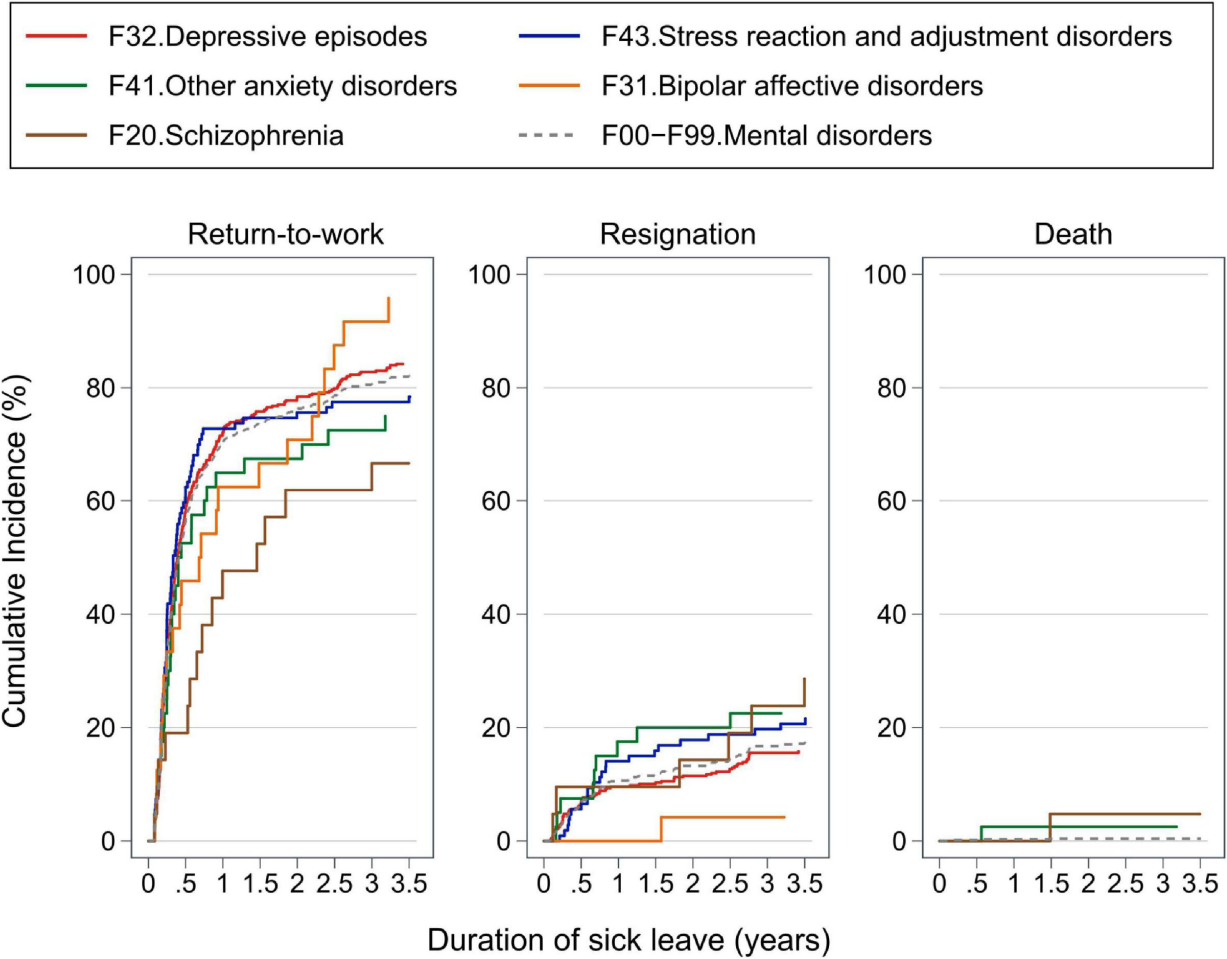
Cumulative incidence of return-to-work, resignation, and death after 30 days or more of sick leave, by major mental disorder

Figure 2 shows the RTW, resignation, and death curves according to ICD-10 codes of mental disorders. The RTW rate increased quickly 1 year after sick leave (1-year RTW rate: depressive episodes, 72.0%; stress reaction and adjustment disorders, 72.8%; other anxiety disorders, 65.0%; bipolar affective disorders, 62.5%; schizophrenia, 47.6%) and flattened after that, except for bipolar affective disorder, which shows bimodality with peaks at 1 and 2.5 years of sick leave. The lowest RTW rate was observed among those with schizophrenia, with a rate of 66.7% at the end of sick leave. A total resignation rate of more than 20% was observed for schizophrenia, stress reaction and adjustment disorders, and other anxiety disorders.

## DISCUSSION

By studying 1,209 first sick-leave episodes lasting a consecutive 30 days or longer in Japanese large-scale private companies, we found that RTW and resignations occurred commonly within 1 year of sick leave regardless of diagnosis, but with different rates depending on the cause of absence. RTW rates are relatively high in circulatory diseases and injuries and low in mental disorders and neoplasms. Death during sick leave was pronounced among long-term absentees with neoplasms. To the best of our knowledge, this is the first report of changes in cumulative incidences of RTW, resignations, and deaths over time, covering major causes of sick leave based on ICD-10 codes in detail with a competing risk analysis.

Among the major causes of sick leave, the present study showed the lowest diagnosis-specific RTW rate for mental disorders throughout the 3.5-year sick-leave period. This is in line with several previous studies that reported a low rate of RTW among those who experienced sickness absence due to mental disorders.^6^^,^^8^^,^^19^

The subgroup analysis of mental disorders showed that the RTW rate was the lowest among those with schizophrenia. For example, the RTW rate at 1 year for schizophrenia was 47.6%, compared to 72.0% for depressive symptoms (F32) and 72.8% for adjustment disorders (F43). Previous studies have also reported this low RTW rate among absentees with schizophrenia. For example, a Dutch study reported that the 1-year RTW rate was 59% for schizophrenia and schizotypal and delusional disorders (F20–F29), while that for neurotic, stress-related, and somatoform disorders was 89%.^6^ A Finnish study of 90 days or more of sick leave or disability pensions reported that the 1-year RTW rate was 41% for those with schizophrenia and 93% for severe stress and adjustment disorders.^11^ Given that schizophrenia is a major cause of long-term sick leave due to a mental disorder, improving the RTW rate among long-term absentees with schizophrenia remains a significant challenge to be addressed.

It is also of note that the RTW curve for bipolar disorder differs from that of other mental disorders. While the RTW rate of bipolar disorder (62.5%) was lower at 1 year after the start of long-term sick leave, the eventual RTW rate was the highest (95.8%) in bipolar disorder. The increase in RTW rate among those who took long-term sick leave due to bipolar disorder seemed to have occurred when their sick-leave benefits were about to expire. More studies are needed to understand this pattern.

Absentees with neoplasms showed a relatively high RTW rate (75.2% by the end of the first year, 80.5% by the end of the second year) in the present study. Previous studies reported similar RTW rates. For example, the full RTW rate for all cancers was 62.3% by 1 year in Japan^9^ and 73% by 2 years in the Netherlands.^10^ We observed a visible death rate (10.6% by 1 year, 16.8% by the end of sick leave) and a quite low resignation rate (1.8% by 1 year, 2.7% by the end of sick leave) among absentees with neoplasms. Since cancer deaths are mainly due to advanced cancer, early cancer detection would further contribute to the improvement of RTW rates and decrease the number of long-term absentees with neoplasms. It is also important to create an environment that makes it easy for those who continue treatment after their return to work to continue working.

The RTW rate for circulatory diseases (I00–I99) was 85.9% within the first year after sick leave. While we are unaware of any studies that examined the RTW rate among those who took sick leave due to circulatory diseases in general, a review article reported that the median RTW rate among 18 longitudinal studies for stroke survivors was 53% at 1 year after diagnosis.^5^ A study in Japan reported that the RTW rate at 1 year for those who were absent from work due to stroke was 62.4%.^20^ For RTW after acute myocardial infarction, the rate 1 year after diagnosis was reported as 55.9% in China,^21^ 91.1% in Denmark,^12^ and 93% in the United States.^22^ It is important to create conditions that make it easier for absentees, particularly those who have suffered the after-effects of cardiovascular disease, to return to work.

The strength of the current study was its high follow-up rate and that it was based on administrative data, which reduced selection bias. The relatively large sample size and sufficiently long follow-up period allowed us to observe the course of sick leave across major diagnostic categories. Additionally, workers in companies with poor benefits may be forced to return to work during recovery for reasons other than health (eg, fear of losing their jobs), but this study tried to minimize such a possibility by including only companies with sufficiently long sick-leave periods in the study. Our study findings may help policy makers and employers realize that 1 year of paid sick leave with sufficient benefit can assist the majority of sick-listed employees in returning to a healthy state and returning to work. Several limitations of this study should be addressed. First, care should be taken when generalizing these results to other study populations. It is possible that generous benefit packages available at the study companies may have led to prolonged sick leave. In addition, the male proportion in the J-ECOH cohort (more than 80%) was higher than the Japanese national average for employees (just under 60%).^23^ Furthermore, since different countries have different social security systems such as sick leave and disability pensions, special attention should be paid to this point when comparing the results of this study with those of surveys conducted in other countries. Second, we defined RTW as when participants started to work again; we do not have information on whether they continued to work after they returned to work. Third, the course of sickness absence may also be affected by the absentee’s work contract (eg, full-time or part-time) and marital status, but these factors could not be taken into account in this study due to the lack of data. Fourth, because of the small sample size of women in this study, it is difficult to discuss gender differences in the results of this study.

In conclusion, RTW and resignations occurred commonly until 1 year after sick leave in the case of long-term sickness absences in major diagnostic groups. This finding has implications for the minimum requirement of the length of paid sick leave. In addition, most long-term absentees with cancer returned to work unless they died, suggesting that promoting early detection of cancer may improve the RTW rate for cancer patients in the Japanese working population.
